# A Perovskite-Based Paper Microfluidic Sensor for Haloalkane Assays

**DOI:** 10.3389/fchem.2021.682006

**Published:** 2021-04-26

**Authors:** Lili Xie, Jie Zan, Zhijian Yang, Qinxia Wu, Xiaofeng Chen, Xiangyu Ou, Caihou Lin, Qiushui Chen, Huanghao Yang

**Affiliations:** ^1^Ministry of Education (MOE) Key Laboratory for Analytical Science of Food Safety and Biology, Fujian Provincial Key Laboratory of Analysis and Detection Technology for Food Safety, College of Chemistry, Fuzhou University, Fuzhou, China; ^2^Department of Neurosurgery, Fujian Medical University Union Hospital, Fuzhou, China; ^3^Fujian Science and Technology Innovation Laboratory for Optoelectronic Information of China, Fuzhou, China

**Keywords:** colorimetric, microfluidic, anion exchange, perovskite, haloalkanes

## Abstract

Detection of haloalkanes is of great industrial and scientific importance because some haloalkanes are found serious biological and atmospheric issues. The development of a flexible, wearable sensing device for haloalkane assays is highly desired. Here, we develop a paper-based microfluidic sensor to achieve low-cost, high-throughput, and convenient detection of haloalkanes using perovskite nanocrystals as a nanoprobe through anion exchanging. We demonstrate that the CsPbX_3_ (X = Cl, Br, or I) nanocrystals are selectively and sensitively in response to haloalkanes (CH_2_Cl_2_, CH_2_Br_2_), and their concentrations can be determined as a function of photoluminescence spectral shifts of perovskite nanocrystals. In particular, an addition of nucleophilic trialkyl phosphines (TOP) or a UV-photon-induced electron transfer from CsPbX_3_ nanocrystals is responsible for achieving fast sensing of haloalkanes. We further fabricate a paper-based multichannel microfluidic sensor to implement fast colorimetric assays of CH_2_Cl_2_ and CH_2_Br_2_. We also demonstrate a direct experimental observation on chemical kinetics of anion exchanging in lead-halide perovskite nanocrystals using a slow solvent diffusion strategy. Our studies may offer an opportunity to develop flexible, wearable microfluidic sensors for haloalkane sensing, and advance the in-depth fundamental understanding of the physical origin of anion-exchanged nanocrystals.

Haloalkanes are an important group of chemical compounds widely used as solvents and reactants in pharmaceutical and chemical industries (Kinani et al., 2016; Daud et al., 2018). Accurate detection and identification of haloalkanes are of great industrial and scientific importance (Leri et al., 2006; Fu et al., 2020; Gul et al., 2020), because many of these compounds exhibit high toxicities to environment and human health, such as carcinogenicity and nephrotoxicity. Over the years, several techniques have been developed for the detection of haloalkanes, such as chromatic, fluorescent indicators, X-ray absorption near-edge structure (XANES) spectroscopy, ultrahigh-resolution mass spectrometry (UHR-MS), and liquid chromatography–mass (LC-MS) (Leri et al., 2006; Roveretto et al., 2019; Fu et al., 2020; Gul et al., 2020; Li et al., 2020; Yin et al., 2021). Despite its importance, precise and rapid quantification of haloalkanes remains a technical challenge. Inherent limitations, such as the bandwidth of recording, restrict the available resolution. Additionally, these technologies are generally limited by their lack of high selectivity. For practical applications, rapid and high-throughput analysis of pollutants are highly desired for on-site testing.

Lead-halide perovskite nanocrystals (NCs) are an emerging class of materials that could be used to achieve fast, sensitive, and selective detection of halides through anion exchange, owing to its unique property of soft and predominantly ionic lattice (Akkerman et al., 2018; Chen et al., 2018; Geng et al., 2018). These materials have been well-developed to be applied in solar cells, light-emitting devices, photodetectors, and photocatalysis, because of their unique facile synthesis, high photoluminescence quantum yields and optical versatility (Huang et al., 2016; Zhou et al., 2016; Kovalenko et al., 2017; Li et al., 2017; Yin et al., 2017). Multicolor photoluminescence emissions can be readily tuned to various wavelengths in the visible spectrum either by adjusting the ratio of halide atoms (Cl, Br, I) or by facile anion exchange (Xing et al., 2014; Protesescu et al., 2015; Wong et al., 2019). Notably, the high mobility of halide anions and the high concentration of halide vacancies result in fast rate during anion exchanging (Parobek et al., 2017; Yoon et al., 2018; Zheng et al., 2018). Direct observation of dynamic process in anion exchanging is still difficult, owing to its fast chemical kinetics, typically within a few seconds (Pan et al., 2018). Although lead-halide perovskite nanocrystals are promising in haloalkanes sensing (Zhu Y. et al., 2019; Li et al., 2021), the fabrication of an on-site testing device is highly desired for low-cost, convenient applications.

Over the past two decades, microfluidic lab-on-a-chip (LOC) technologies have increasingly emerged as a powerful tool for point of care testing, by taking the advantages of low sample consumption, low-cost production, and high-throughput rapid analysis (Xie et al., 2019; Miller et al., 2020). In particular, paper-based microfluidic sensors are attractive to perform the real-time measurements *in-situ* by designing an on-demand pattern of the channels (Cate et al., 2015). This makes paper-based microfluidic devices of particular interest in testing analyses when combined with luminescence nanocrystals. Here, we demonstrate a paper-based multichannel microfluidic platform for detection of haloalkanes through anion exchanging in perovskite nanocrystals. We show a direct observation of the chemical kinetics of anion exchanging between perovskite nanocrystals and haloalkanes, as a result of a slow solvent diffusion. We also demonstrate that the lead-halide perovskite nanocrystals-based paper microfluidic sensor is affordable to achieve a fast, convenient analysis of haloalkanes based on colorimetric sensing.

To validate our hypothesis, we synthesized lead-halide perovskite nanocrystals by reacting Cs-oleate precursors with PbX_2_ (X = Cl, Br or I), using a hot-injection solution strategy at 160°C. Transmission electron microscopy (TEM) images indicate the well-defined cubic morphologies of the as-synthesized CsPbCl_3_, CsPbBr_3_, and CsPbBr_0.5_/I_2.5_ perovskite nanocrystals (Figure 1A), with an average size of about 11 nm (Supplementary Figure 1). X-ray diffraction (XRD) measurements were conducted to confirm the cubic phase of the perovskite nanocrystals (Figure 1B). Figure 1C shows photoluminescence spectra of the as-synthesized perovskite nanocrystals, which display high photoluminescence yields and color-tunable emissions in blue, green, and red regions. The highly ionic nature of perovskite nanocrystals allows for readily engineering their optical emissions through facile and rapid anion exchange. Such unique capability can be employed to achieve rapid detection of halide compounds through anion exchanging.

**Figure 1:**
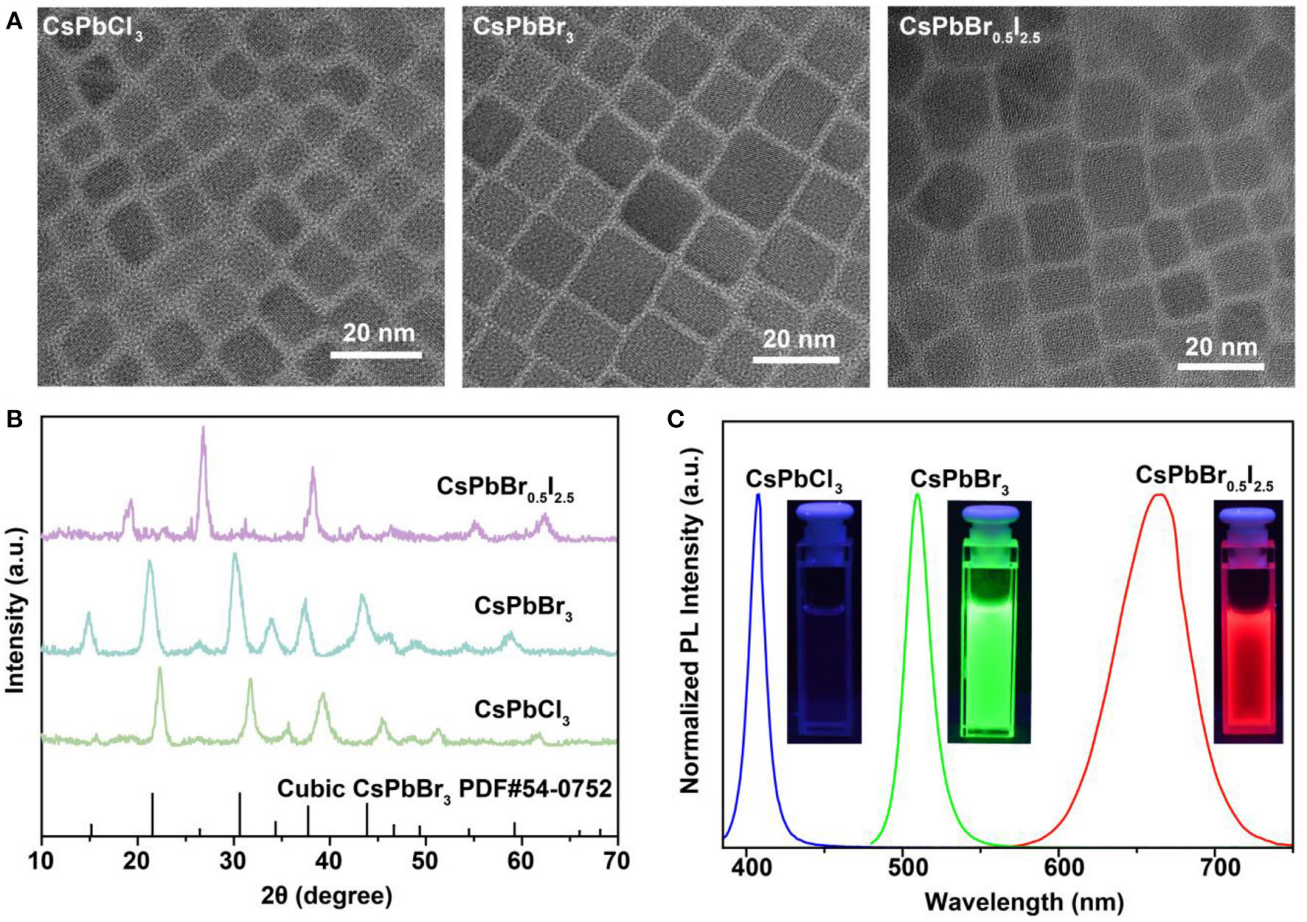
Characterization and spectroscopy study of as-synthesized CsPbX_3_ (X = Cl, Br, or I) perovskite nanocrystals. **(A)** TEM images of the as-synthesized cubic-phase perovskite nanocrystals. The samples are CsPbCl_3_, CsPbBr_3_, and CsPbBr_0.5_I_2.5_ nanocrystals. **(B)** XRD patterns of the as-synthesized perovskite nanocrystals. All peaks are consistent with the cubic-phase CsPbBr_3_ structure [Joint Committee on Powder Diffraction Standards file (PDF) number 54-0752]. **(C)** Fluorescence spectra of the perovskite nanocrystals under 365-nm UV excitation. The insets show photographs of the samples under 365-nm UV excitation.

To assess the feasibility of the perovskite nanocrystals as nanoprobes for colorimetric sensing of haloalkanes, we used CH_2_Br_2_ as an analyte sample by reacting with CsPbCl_3_ and CsPbBr_0.5_I_2.5_ nanocrystals. Our experimental results indicated that their luminescence emission colors were readily shifted as a result of the anion exchanging process (Figure 2A; Supplementary Figures 2, 3). Note that the passivation of CsPbBr_3_ nanocrystals with bromide-enriched CH_2_Br_2_ molecules can enhance their luminescence emission, owing to the efficiently reduced surface quenching defects (Supplementary Figure 4). Moreover, the use of TOP or UV illumination is capable of accumulating the anion exchanging rates (Supplementary Figures 5, 6). The wavelength shift in the photoluminescence emission spectra is attributed to the change of bandgaps of the perovskite nanocrystals as a result of the exchange of bromide with Cl or I in the lattice of CsPbBr_3_ and CsPbBr_0.5_I_2.5_ nanocrystals. The changes in emission color are conveniently visual for colorimetric sensing of samples, as indicated in the CIE chart (Figure 2B). Furthermore, we examined the suitability of using this method to achieve a quantitative detection of CH_2_Cl_2_ and CH_2_Br_2_ samples through measuring the wavelength shift of photoluminescence from perovskite nanocrystals, as shown in Figures 2C,E. With the increase in the analyte concentrations, the photoluminescence emission wavelength of the CsPbBr_3_ nanocrystals in solution was shifted from 510 to 460 nm, with a linear equation of *y* = 0.69x + 510.2 (y is the wavelength and x is the sample concentration) and a detection limit of 4.12 mg/ml for CH_2_Cl_2_. Similarly, we demonstrated a detection limit of 0.29 mg/ml for CH_2_Br_2_ sensing (Figures 2D,F). These results suggested that our method was sufficient for qualitative and quantitative analysis of halide compounds.

**Figure 2:**
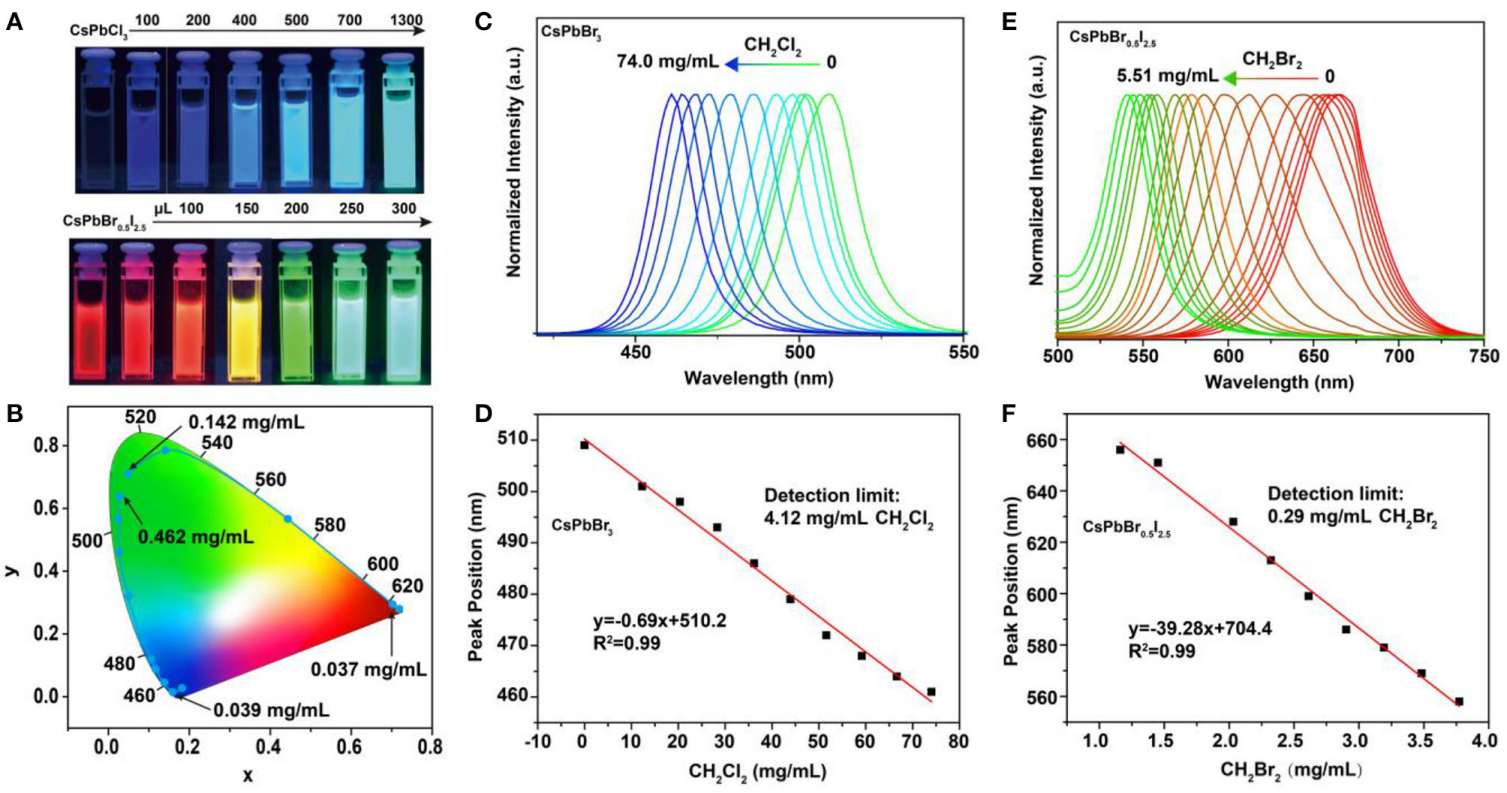
Detection of haloalkanes using perovskite nanocrystals based on anion exchanging. **(A)** Luminescence color changing of perovskite nanocrystals when adding various CH_2_Br_2_ samples. **(B)** CIE (Commission International de l'Eclairage) chromaticity coordinates from the fluorescence spectra of perovskite nanocrystals reacting with various CH_2_Br_2_ samples. **(C,E)** Normalized emission spectra of CsPbBr_3_ and CsPbBr_0.5_I_2.5_ nanocrystals, as a function of the CH_2_Cl_2_ and CH_2_Br_2_ amounts, respectively. **(D,F)** A linear relation between the analytes (CH_2_Cl_2_ and CH_2_Br_2_) concentrations and luminescence emission peaks of perovskite nanocrystals.

We carried out experiments to fabricate a paper-based microfluidic device for achieving on-site detection of haloalkane. Figure 3a shows the design of a six-channel paper microchip containing three types of perovskite nanocrystals. The detection of the targeted haloalkane samples was implemented by diffusing them into the perovskite nanocrystals via anion exchange-mediated reaction. In a typical experiment, we deposited the perovskite nanocrystals of CsPbCl_3_, CsPbBr_3_, and CsPbBr_0.5_I_2.5_ in the microchannels which emitted deep blue, green, and red fluorescence under a 365-nm excitation (Figure 3b). The CH_2_Cl_2_ sample was added to the central region of the designed paper microfluidic device. Our experiments indicated that the green fluorescence of CsPbBr_3_ nanocrystals was quickly changed into blue as a result of anion exchanging-mediated reaction under UV illumination. This suggested that our design is suitable for a fast detection of CH_2_Cl_2_ sample by convenient colorimetric sensing (Figure 3c). In a parallel set of experiments, we demonstrated that the device can also be employed to qualitatively detect CH_2_Br_2_ sample through monitoring the fluorescence color change of CsPbCl_3_ and CsPbBr_0.5_I_2.5_ channels (Figure 3d). We further demonstrated that our method is suitable for the detection of a mixed sample containing both CH_2_Cl_2_ and CH_2_Br_2_ (Figure 3e; Supplementary Figure 7). The reaction mechanism for the haloalkane sensing is illustrated in Figure 3f, an anion exchange process with CsPbX_3_ (X = Cl, Br, or I) nanocrystals. Haloalkane molecules, such as CH_2_Br_2_, were introduced *in-situ* near the surface of perovskite nanocrystals to implement the anion-exchange reaction, upon either an addition of nucleophilic trialkyl phosphines (TOP) or a UV-photon-induced electron transfer from CsPbX_3_ nanocrystals (Figure 3f). The change of halide composition after the anion exchanging leads to continuous changes of the bandgap, as well as the absorption and emission spectra in the perovskite nanocrystals (Figure 3g).

**Figure 3:**
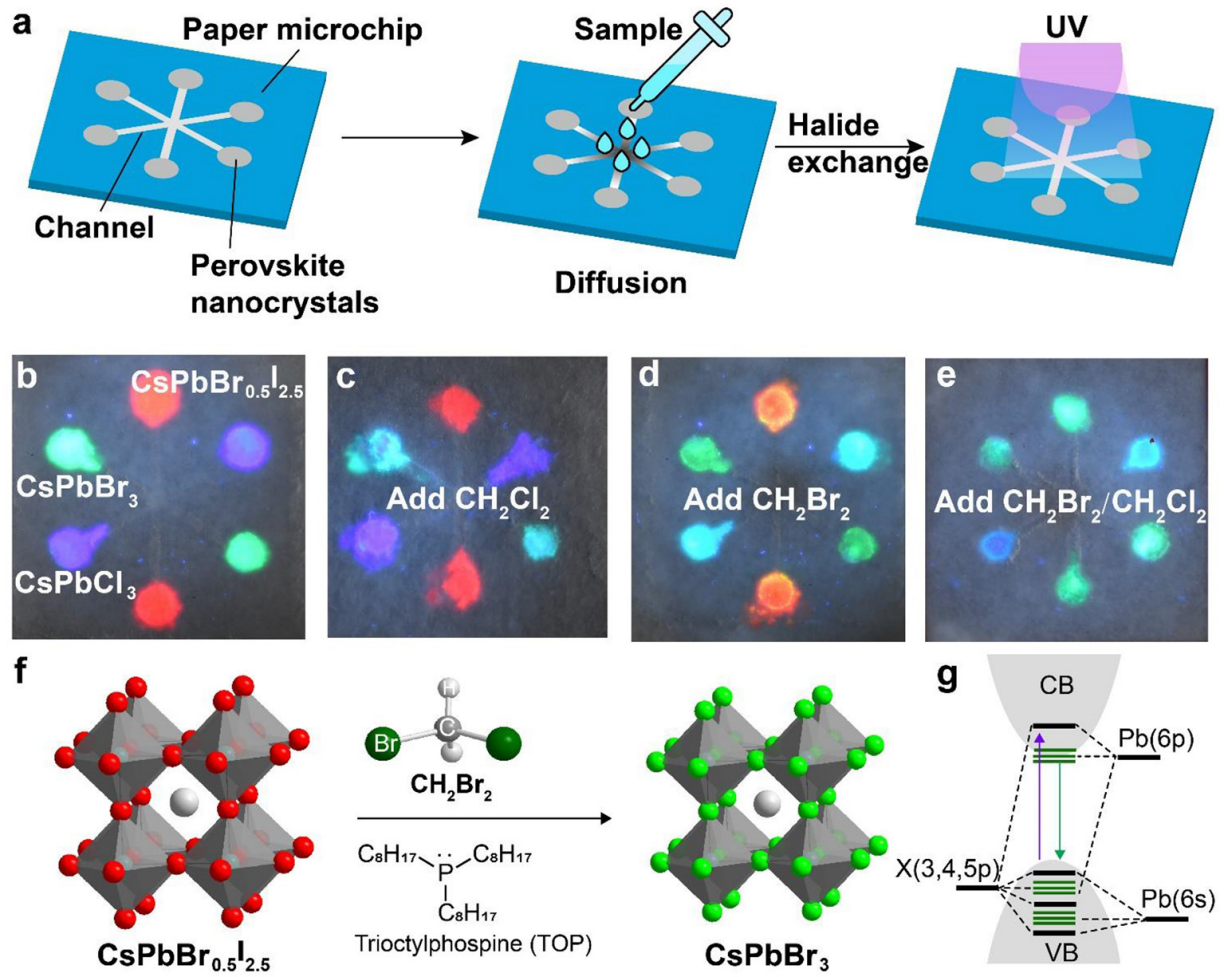
Schematic of a perovskite microfluidic platform for on-site detection of haloalkane. **(a)** Design of a paper-based microfluidic chip for multichannel detection of haloalkane. The perovskite nanocrystals were firstly filled in the paper microchip, and then the analyte samples were dropped and diffused along the microchannel. Upon UV irradiation, on-site sensing can be implemented as a result of a fast anion exchange reaction. **(b)** Luminescence image of a paper-based microfluidic device filled with perovskite nanocrystals (CsPbCl_3_, CsPbBr_3_, and CsPbBr_0.5_I_2.5_). The photograph was taken under a 365-nm UV excitation. **(c–e)** Colorimetric sensing of CH_2_Cl_2_ and CH_2_Br_2_, using the paper-based microfluidic device from **(b)**. The photographs were taken under a 365-nm UV excitation. **(f)** Reaction mechanism of a nucleophile (TOP)-mediated anion exchange between perovskite nanocrystals and halides. **(g)** Schematic of the electronic structures for perovskite nanocrystals.

The physical process for haloalkane detection was investigated by monitoring the chemical kinetics of the anion-exchanged reaction between perovskite nanocrystals and haloalkane. A solvent mixture of CH_2_Br_2_ and cyclohexane containing nucleophilic TOP was homogeneously mixed with CsPbBr_0.5_I_2.5_ nanocrystals. Under UV illumination, we observed a gradual change in the photoluminescence emission color, as a function of halide exchanging time. Figure 4A shows the green photoluminescence emission via anion exchange for 1 min. In a parallel experiment, 10 μL of CsPbBr_0.5_I_2.5_ perovskite nanocrystals were dropped into a mixture of CH_2_Br_2_ and cyclohexane containing nucleophilic TOP. Intriguingly, a colorful rainbow-based photoluminescence emission was observed under UV illumination (Figure 4B). The gradual transition in photoluminescence emission color from red to green was attributed to the slow diffusion of perovskite nanocrystals into the solvent. A similar result was also obtained by diffusing CsPbCl_3_ nanocrystals into the mixture of CH_2_Br_2_ and cyclohexane (Figure 4C; Supplementary Figure 8). The direct experimental observation on the chemical kinetics of anion exchange in perovskite nanocrystals offers a powerful strategy for in-depth understanding of the physical process of anion exchanging in perovskite nanocrystals.

**Figure 4:**
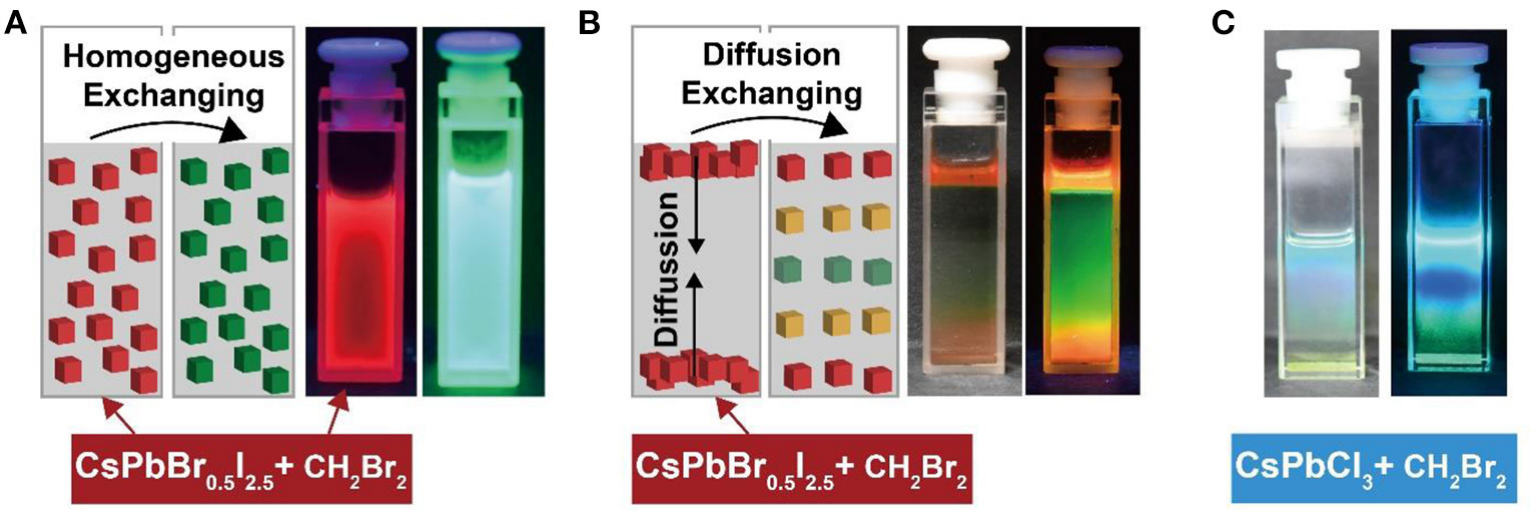
Direct observation of chemical kinetics of anion exchanging between perovskite nanocrystals and haloalkanes through slow solvent diffusion. **(A)** Homogeneous anion exchanging between CsPbBr_0.5_I_2.5_ and CH_2_Br_2_. The luminescent images were recorded before and after anion exchanging under a 365-nm UV illumination. **(B)** Diffusion anion exchanging between CsPbBr_0.5_I_2.5_ and CH_2_Br_2_. **(C)** Diffusion anion exchanging between CsPbCl_3_ and CH_2_Br_2_.

In summary, we have developed a perovskite-based paper microfluidic sensor for detection of haloalkanes through anion exchanging. Our experimental results demonstrated that, by combing with perovskite nanocrystals, the paper-based multichannel microfluidic device offers a low-cost, high-throughput and convenient platform for fast colorimetric sensing of haloalkanes. The direct experimental observation on chemical kinetics of slow diffusion-mediated anion exchanging in perovskite nanocrystals may be valuable for a fundamental understanding on the materials synthesis and optical properties of perovskite nanocrystals for various applications, such as X-ray imaging and photocatalysis (Chen et al., 2018; Zhu X. et al., 2019; Ou et al., 2021). Future work can be devoted to designing the flexible microfluidic sensors suitable for achieving on-site qualitative and quantitative analysis of haloalkane and for both visual and instrumental readout.

## Data Availability Statement

The original contributions presented in the study are included in the article/Supplementary Material, further inquiries can be directed to the corresponding authors.

## Author Contributions

LX, ZY, CL, QC, and HY contributed to the conception and design of the experiments. LX, JZ, and ZY contributed to the materials synthesis, sample testing, and wrote the draft of the manuscript. QW, XC, and XO performed the data analysis. CL, QC, and HY wrote the manuscript. All authors contributed to approving the submitted version.

## Conflict of Interest

The authors declare that the research was conducted in the absence of any commercial or financial relationships that could be construed as a potential conflict of interest.
