# Supplementary material for: A Perovskite-Based Paper Microfluidic Sensor for Haloalkane Assays
Source: Front Chem. 2021 Apr 26;9:682006. doi: 10.3389/fchem.2021.682006 (PMC8107377; doi:10.3389/fchem.2021.682006)
Supplement: Supplementary file 1 [file Presentation_1.pdf]

Supplementary Materials

A perovskite-based paper microfluidic sensor for haloalkane assays

Lili Xie,[1] Jie Zan,[1] Zhijian Yang,[1] Qinxia Wu,[1] Xiaofeng Chen,[1] Xiangyu Ou,[1] Caihou Lin,[2] Qiushui Chen,[1,3*] & Huanghao Yang[1,3*]*

[1]*MOE Key Laboratory for Analytical Science of Food Safety and Biology, Fujian Provincial Key Laboratory of Analysis and Detection Technology for Food Safety, College of Chemistry, Fuzhou University, Fuzhou 350002, China.*
[2]*Department of Neurosurgery, Fujian Medical University Union Hospital, Fuzhou, Fujian 350001, China.*
[3]*Fujian Science & Technology Innovation Laboratory for Optoelectronic Information of China, Fuzhou 350108, P. R. China.*

[*]Corresponding author. E-mail: lincai76@163.com (C.L.); qchen@fzu.edu.cn (Q.C.); hhyang@fzu.edu.cn (H.Y.)

Chemicals

Lead(II) iodide (PbI_2, 99.99%), lead(II) bromide ($PbBr_2$, 99.99%), lead(II) chloride ($PbCl_2$, 99.99%), Cesium carbonate (Cs_2CO_3, 99.9%), trioctylphospine (TOP), cyclohexane (chromatography grade 99.9%), oleic acid (technical grade 90%), oleylamine (technical grade 70%), 1-octadecene (ODE, technical grade 90%), dichloromethane (CH_2Cl_2), and dibromomethane (CH_2Br_2) were obtained from Sigma-Aldrich. Sylgard® 184 silicone elastomer kit for polydimethylsiloxane (PDMS) coating was obtained from Dow Corning. Chromatography paper (100 μm thick) was purchased from Whatman. All chemicals were used without further treatment.

Synthesis of perovskite nanocrystals

Cs-oleate precursor was prepared by adding Cs_2CO_3 (0.4 g), octadecene (ODE; 15 mL) and oleic acid (OA; 1.25 mL) to a 50-ml 2-neck flask. The suspension was heated to 120 ^oC under stirring in vacuum for 30 min. The reaction was further heated to 150 ^oC to ensure a complete reaction of Cs_2CO_3 and OA to form the Cs-oleate precursor solution. Perovskite nanocrystals of $CsPbX_3$ (X = Cl, Br, or I) were synthesized by a previously reported method. PbX_2 (0.36 mmol), oleylamine (1.0 mL), ODE (10 mL), and oleic acid (1.0 mL) were injected to a 50-ml 2-neck flask. The suspension was heated to 120 ^oC under vigorous stirring in vacuum for 30 min. Subsequently, the reaction was treated by purging with nitrogen and vacuum suction, and then heated to 160 ^oC for the complete dissolution of the PbX_2 precursors. 1 mL of hot Cs-oleate precursor was injected into the above-mentioned mixture. After 5 s, the reaction was terminated by transferring the flask into an ice bath. The $CsPbX_3$ nanocrystals were collected by centrifugation at 10000 rpm for 10 min and stored in 4-mL cyclohexane.

Physical characterization

TEM characterization was performed by a Tecnai G2 F20 S-TWIN microscope (FEI Nano Ports), operating at an accelerating voltage of 200 kV. X-ray diffraction analysis was carried out by an X-ray powder diffractometer (DY5261/Xpert3) over an angular range of 5–90°. Luminescent spectra were taken by an Edinburgh Fls980 fluorescence spectrophotometer (Edinburgh Instruments). Absorption spectra were recorded using a UV-Vis spectrophotometer (UV-2450, Shimadzu, Japan). Colorimetric

imaging was performed with a digital camera (Nikon) in an all-manual mode. All spectra were obtained under ambient conditions.

Fabrication of paper microfluidic chips

In a typical procedure, a pattern of six-channel packaging tapes was designed using the Adobe Illustrate software, and printed out by a laser cutting. The patterned tape with six-channels was attached to a chromatography paper. A premixed PDMS prepolymer and its curing agent (10:1 by mass) were degassed, coated on the chromatography paper, and then cured in an 80 °C oven for 10 min. After that, the packaging tape was removed from the chromatography paper, and a six-channel paper microfluidic chip was produced for further experiments.

Haloalkane sensing through anion exchange

A solution (50 mg/mL) of perovskite nanocrystals was dropped into the cyclohexane. Haloalkanes (CH_2Cl_2, or CH_2Br_2) with various concentrations were added to the perovskite nanocrystals. 50 μL of trioctylphospine (TOP) was used to accelerate the anion exchange with perovskite nanocrystals. For detection of haloalkanes on the paper microfluidic chip, 200 μL of perovskite nanocrystals containing 20 μL of TOP were dropped onto the microchips. The haloalkanes were dropped onto the microchip, and diffused along with the microchannels for anion exchange reaction. After the color change of perovskite nanocrystals was observed, luminescence spectra of the perovskite nanocrystals were measured by a spectrometer under 365-nm UV excitation.

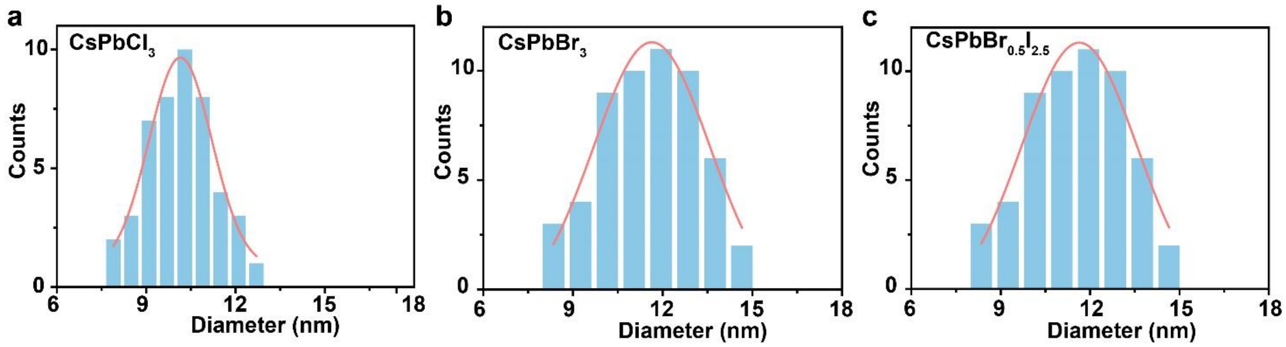

Figure S1. Size distributions of the as-synthesized perovskite nanocrystals. These samples are $CsPbCl_3$ (a), $CsPbBr_3$ (b), and $CsPbBr_{0.5}I_{2.5}$ (c).

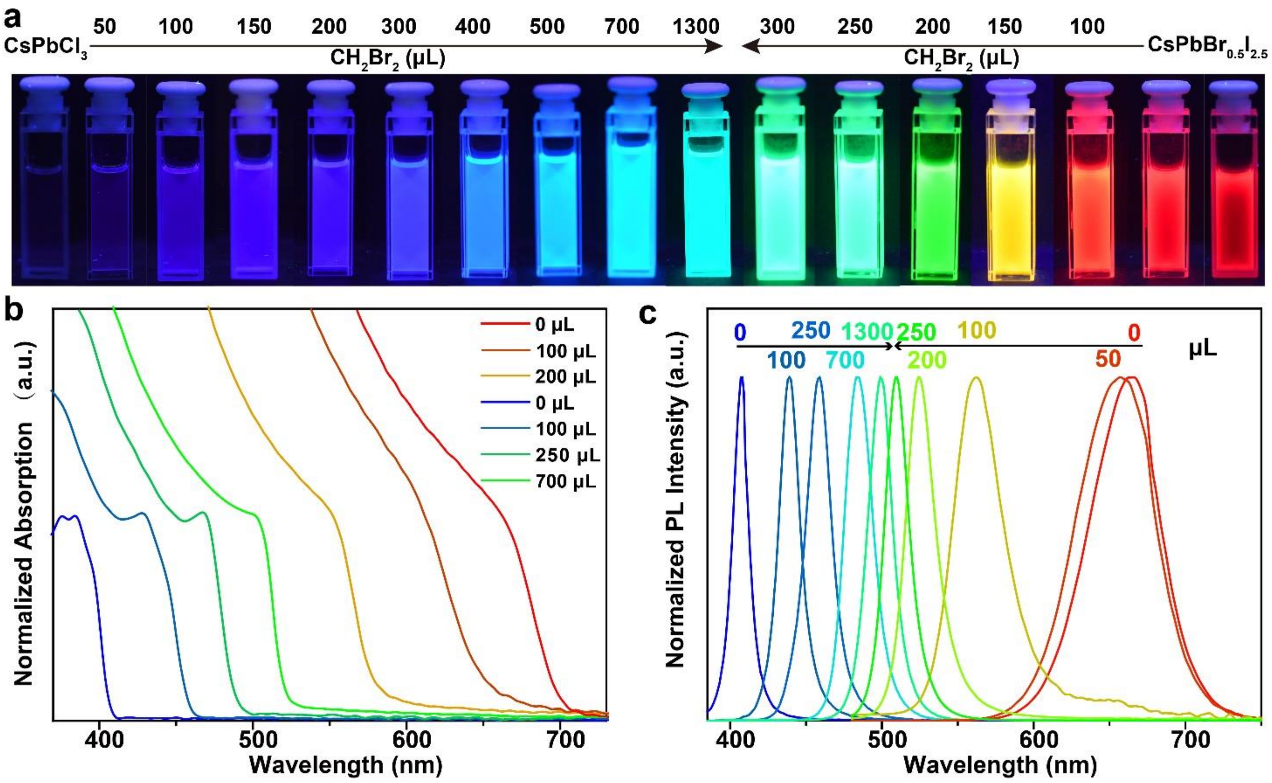

Figure S2 | Optical characterization of perovskite nanocrystals as a function of CH_2Br_2 amounts. (a) Luminescence emission of $CsPbCl_3$ and $CsPbBr_{0.5}I_{2.5}$ in cyclohexane by anion exchanging with varying CH_2Br_2 amounts under 365-nm UV illumination. (b) CH_2Br_2 amount dependence of absorption spectrum for $CsPbCl_3$ and $CsPbBr_{0.5}I_{2.5}$ in cyclohexane. (c) CH_2Br_2 amount dependence of emission spectrum for $CsPbCl_3$ and $CsPbBr_{0.5}I_{2.5}$ in cyclohexane.

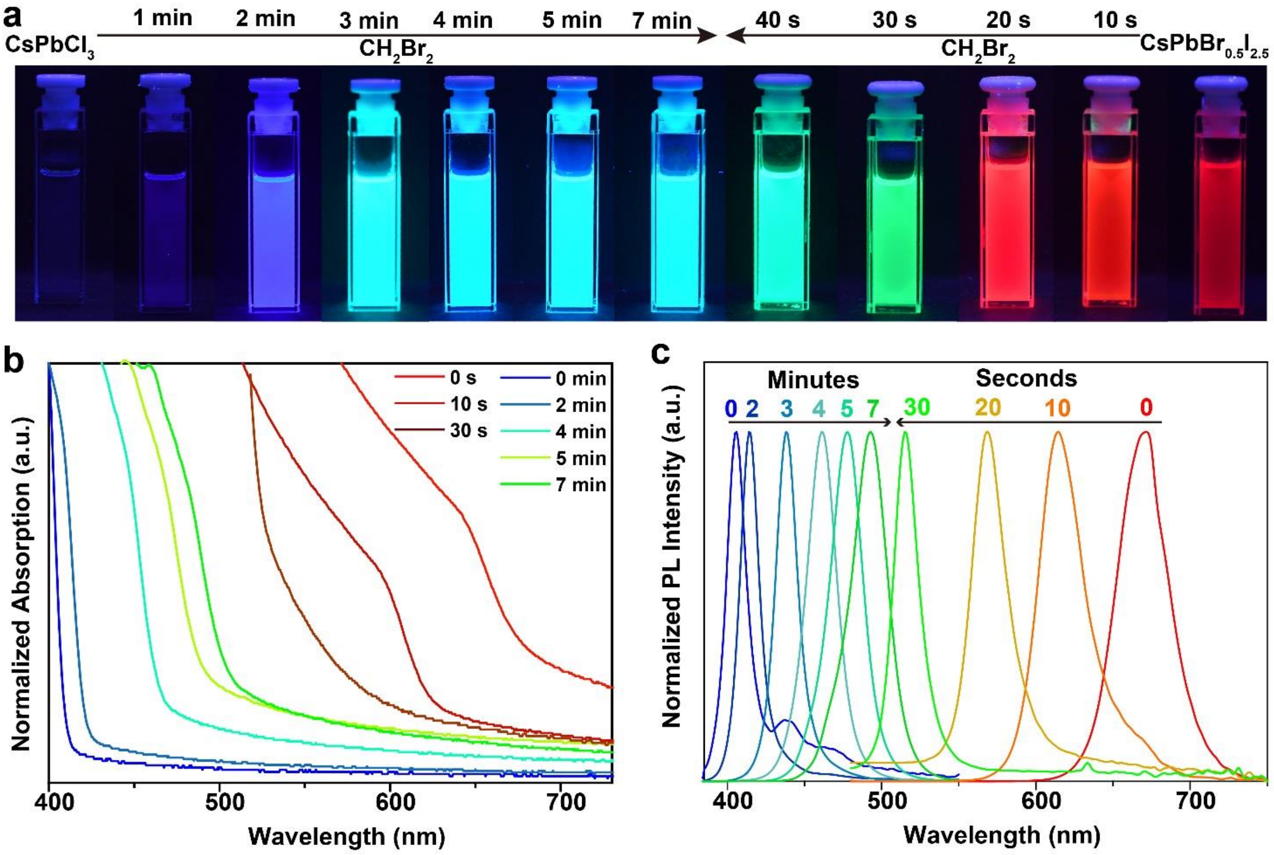

Figure S3 | Optical characterization of perovskite nanocrystals as a function of CH_2Br_2 exchanging time. (a) Luminescence emission of $CsPbCl_3$ and $CsPbBr_{0.5}I_{2.5}$ in cyclohexane by anion exchanging with CH_2Br_2 as a function of exchanging time under an Xe-lamp illumination. (b) Time dependence of absorption spectrum for $CsPbCl_3$ and $CsPbBr_{0.5}I_{2.5}$ in cyclohexane/CH_2Br_2. (c) Time dependence of emission spectrum for $CsPbCl_3$ and $CsPbBr_{0.5}I_{2.5}$ in cyclohexane/CH_2Br_2.

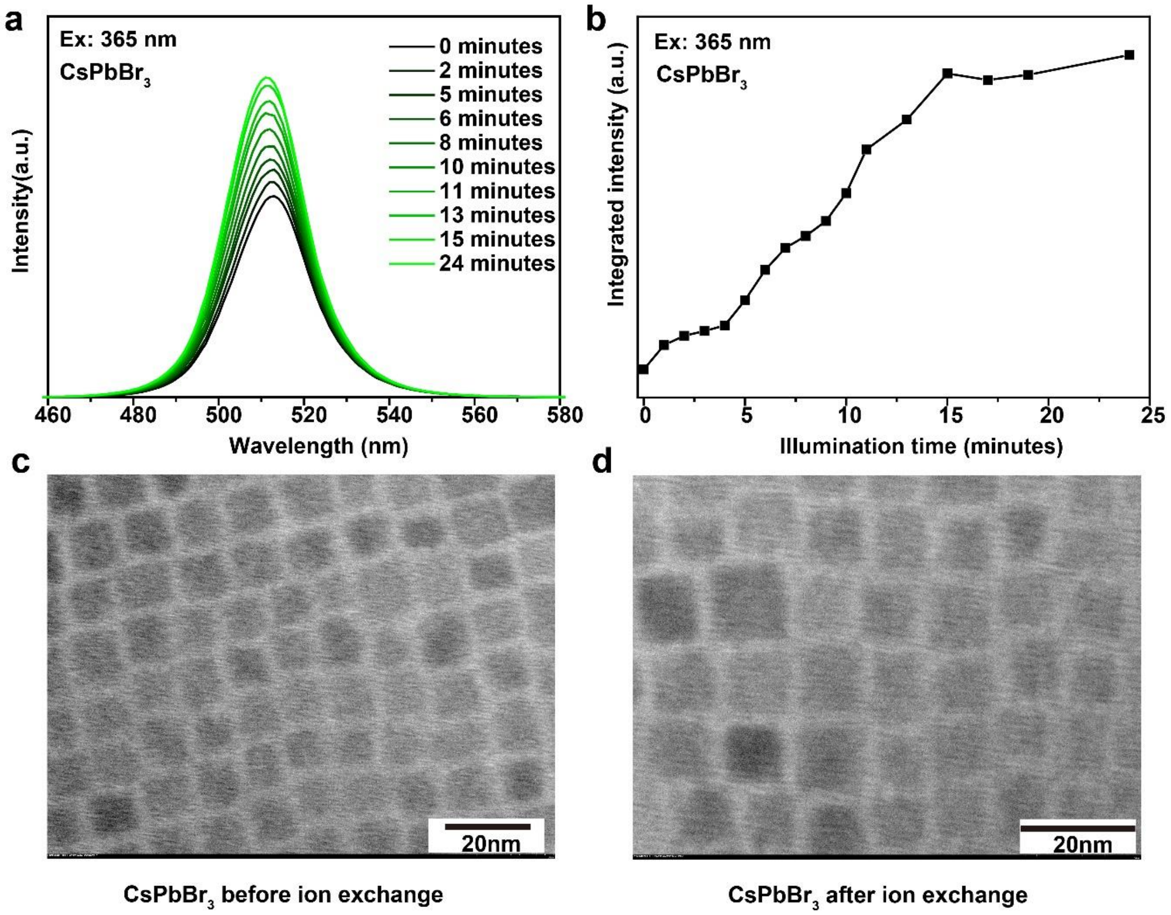

Figure S4 | Changes to the luminescence emission and morphology of $CsPbBr_3$ nanocrystals exposed to varying amounts of CH_2Br_2. (a) Luminescence emission spectra of $CsPbBr_3$ nanocrystals in CH_2Br_2/cyclohexane as a function of time. (b) Luminescence emission intensities of $CsPbBr_3$ nanocrystals in CH_2Br_2/cyclohexane as a function of time. (c) TEM image of pristine $CsPbBr_3$ nanocrystals, showing a cubic shape. (d) TEM image of $CsPbBr_3$ nanocrystals after anion exchanging with CH_2Br_2.

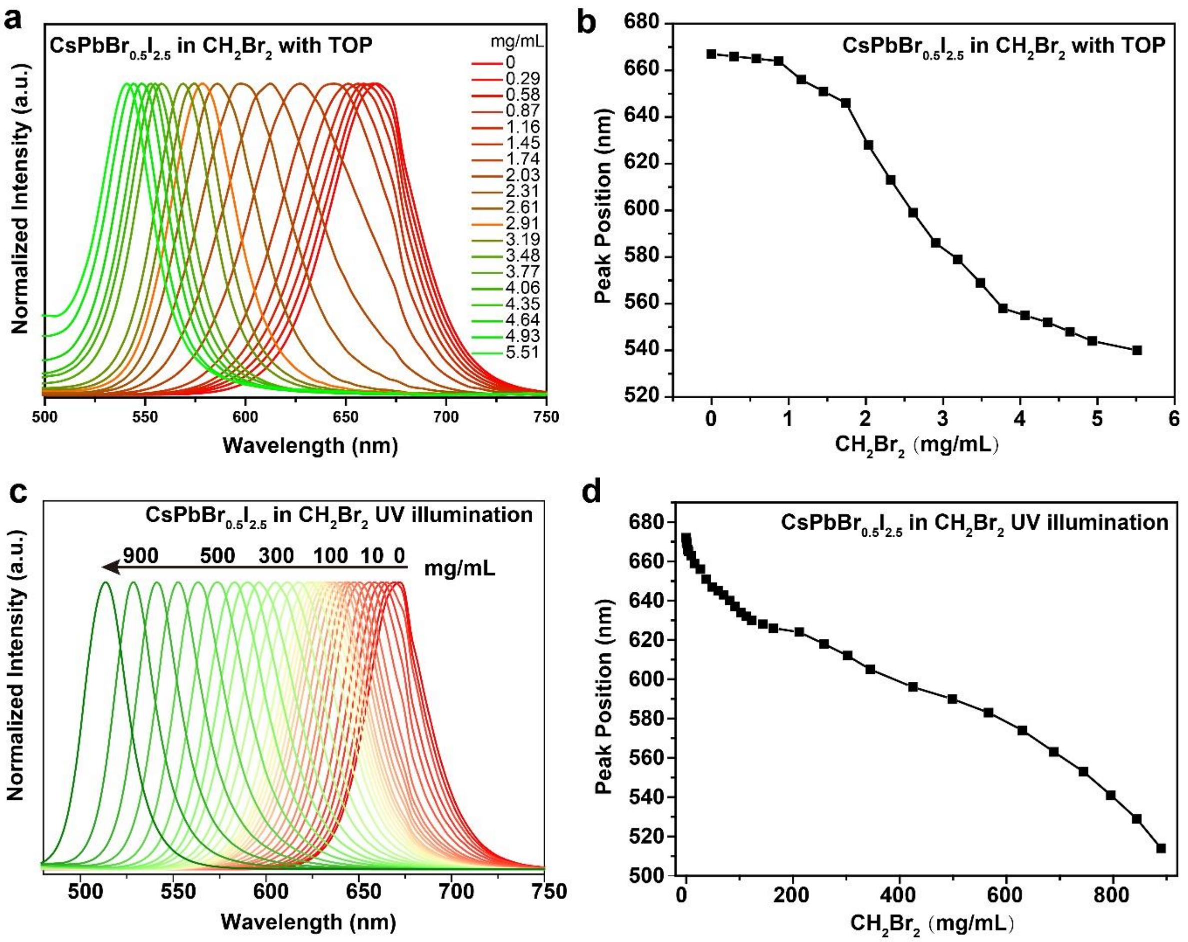

Figure S5 | Detection of CH_2Br_2 using $CsPbBr_{0.5}I_{2.5}$ nanocrystals with the addition of TOP and under UV illumination. (a) Luminescence emission spectra of $CsPbBr_3$ nanocrystals as a function of the amount of the added CH_2Br_2 with TOP. (b) Emission wavelength shift of $CsPbBr_{0.5}I_{2.5}$ nanocrystals as a function of the amount of the added CH_2Br_2 with TOP. (c) Luminescence emission spectra of $CsPbBr_{0.5}I_{2.5}$ nanocrystals upon UV illumination, as a function of the amount of CH_2Br_2 added. (d) Emission wavelength shift of $CsPbBr_{0.5}I_{2.5}$ nanocrystals upon UV illumination, as a function of the amount of CH_2Br_2 added.

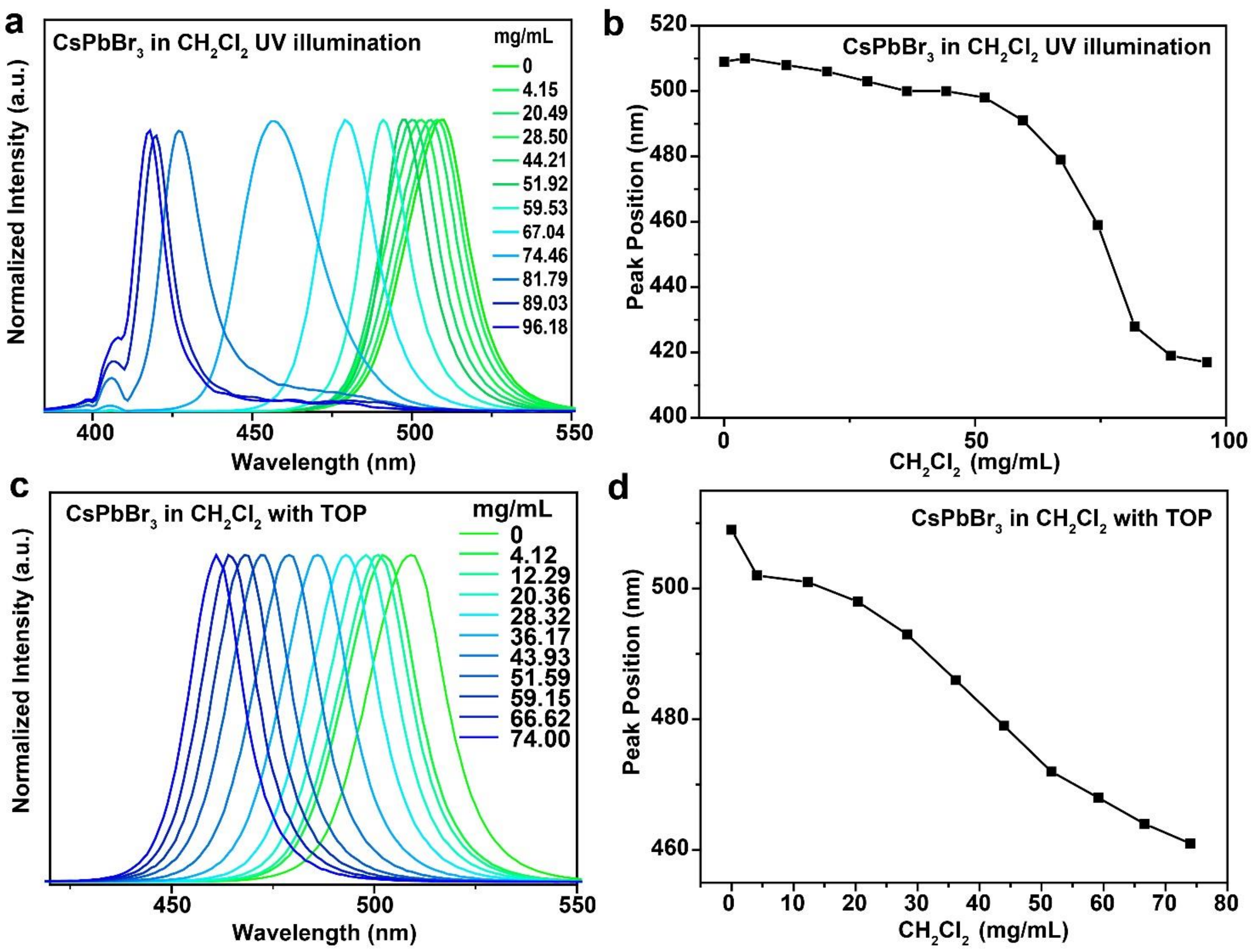

Figure S6 | Detection of CH_2Cl_2 using $CsPbBr_3$ nanocrystals with the addition of TOP and under UV illumination. (a) Luminescence emission spectra of $CsPbBr_3$ nanocrystals under UV illumination, as a function of the amount of CH_2Cl_2 added. (b) Emission wavelength shift of $CsPbBr_3$ nanocrystals under UV illumination, as a function of the amount of CH_2Br_2 added. (c) Luminescence emission spectra of $CsPbBr_3$ nanocrystals as a function of the amount of the added CH_2Cl_2 with TOP. (d) Emission wavelength shift of $CsPbBr_3$ nanocrystals as a function of the amount of the added CH_2Cl_2 with TOP.

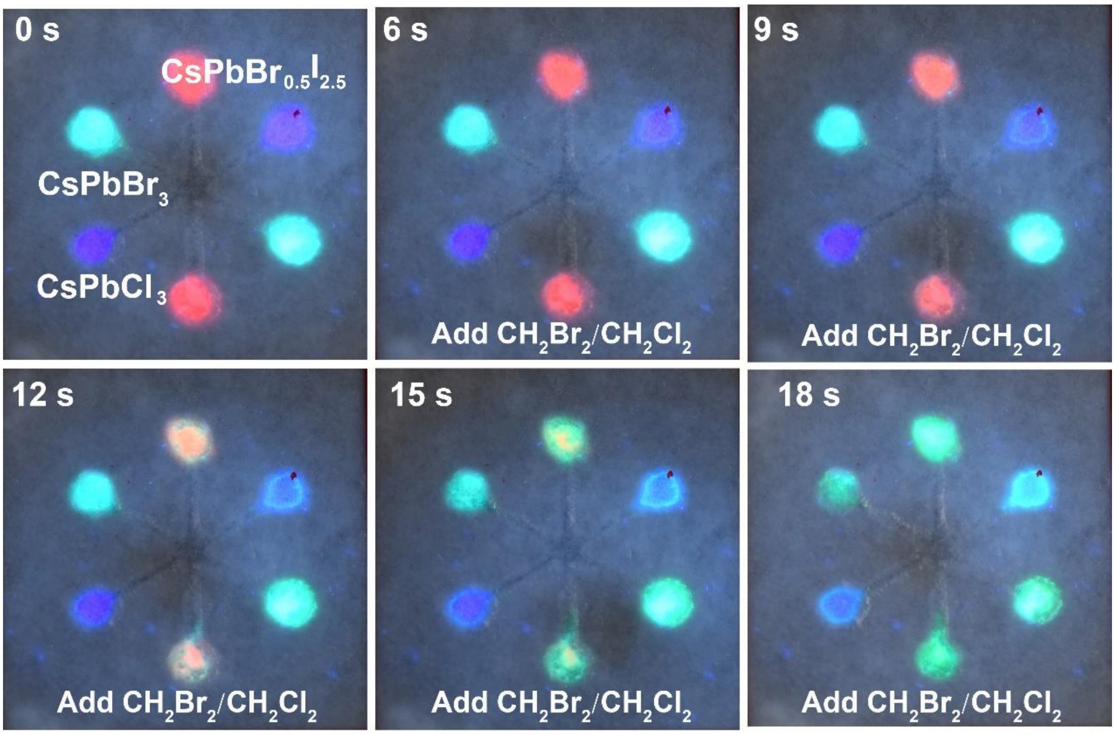

Figure S7 | Luminescence images of perovskite nanocrystals exchanged with CH_2Cl_2/CH_2Br_2 on a paper-based microfluidic device as a function of reaction time.

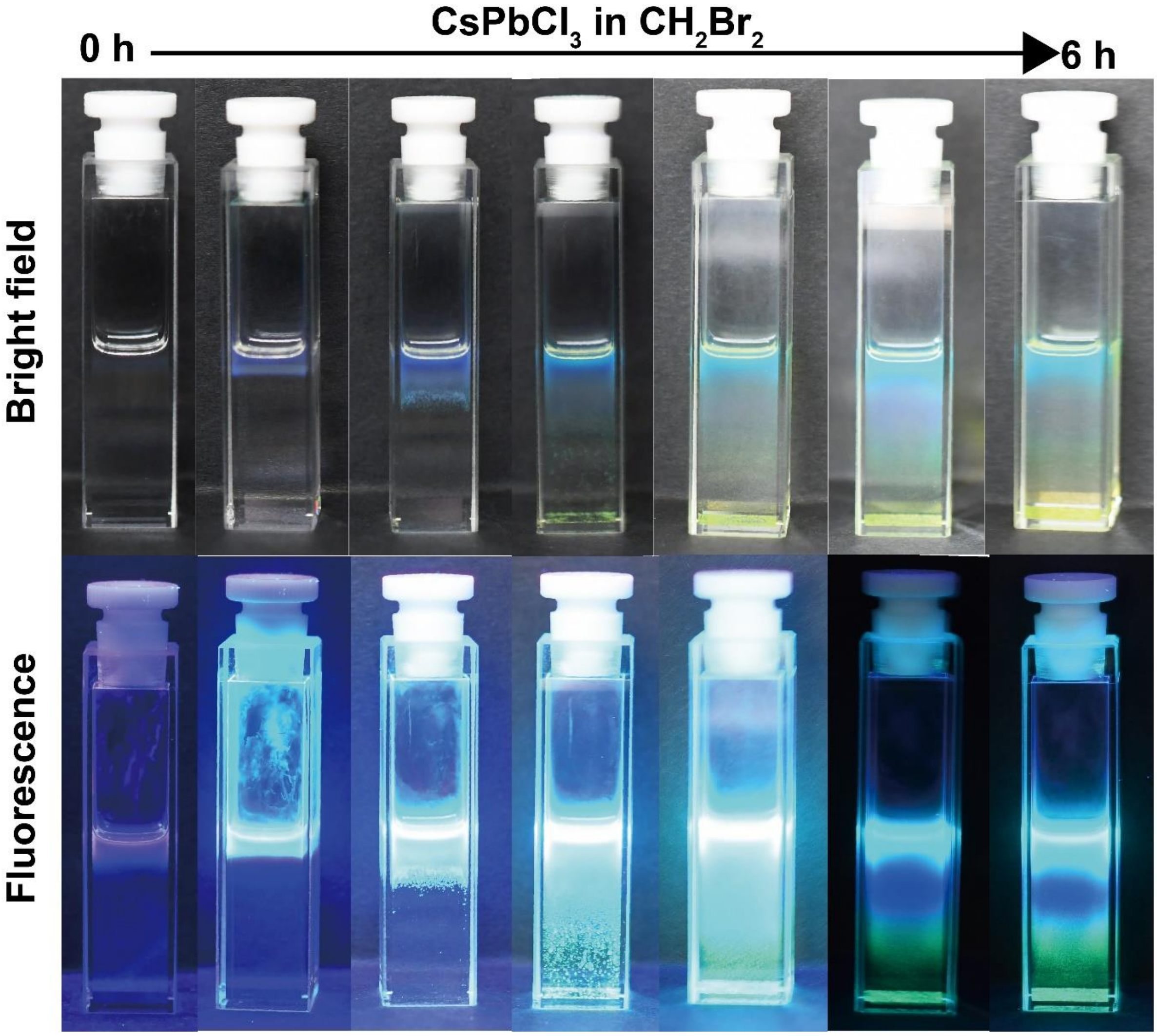

Figure S8 | Bright field and luminescence images of $CsPbCl_3$ nanocrystals exchanged with CH_2Br_2 as a function of reaction time.
